# Prevalence of intestinal parasites and associated risk factors among HIV/AIDS patients with pre-ART and on-ART attending dessie hospital ART clinic, Northeast Ethiopia

**DOI:** 10.1186/1742-6405-10-7

**Published:** 2013-02-25

**Authors:** Assefa Missaye, Mulat Dagnew, Abebe Alemu, Agersew Alemu

**Affiliations:** 1 Dessie regional laboratory, Amhara regional state, Northeast Ethiopia; 2 Department of Medical Microbiology, School of Biomedical and Laboratory Sciences, College of Medicine and Health Sciences, University of Gondar, Gondar, Ethiopia; 3 Department of Medical Parasitology, School of Biomedical and Laboratory Sciences, College of Medicine and Health Sciences, University of Gondar, Gondar, Ethiopia

**Keywords:** Intestinal parasite, ART, CD4

## Abstract

**Background:**

Intestinal parasites are a major concern in most developing countries where HIV/AIDS case are concentrate and almost 80% of AIDS patients die of AIDS-related infections. In the absence of ART, HIV/AIDS patients in developing countries unfortunately continue to suffer from the consequences of opportunistic parasites. But this prevalence has dramatically decreased in countries where antiretroviral agents are widely available. Therefore, the aim of this study was to assess the prevalence of intestinal parasite and risk factor among pre- ART and on ART adult HIV/ AIDS patients attending ART clinic in Dessie hospital.

**Methods:**

A comparative cross-sectional study was conducted among pre-ART and on ART adult HIV/AIDS patients of Dessie Hospital. A total of 272 (136 from each group) study subjects were selected by using systematic random sampling. Stool sample was collected and processed using direct wet mount, formol-ether concentration technique and modified Ziehl-Neelson staining techniques. A structured questionnaire was used to collect data on Sociodemographic & associated risk factors. Data was entered and analyzed by using SPSS 16 software and logistic regressions were applied to assess any association between explanatory factors and outcome variables.

**Results:**

The overall prevalence of IP in pre-ART and on-ART was 39% and 17.6%, respectively with significant decrease of intestinal parasite in the ART era (p < 0.001). All *Cryptosporidium* spps infections were found in the pre-ART patients and significantly associated for lower CD4 <200cells/mm^3^. Absence of toilet (AOR = 7.57; 95% CI = 1.3,44.22), source of water (AOR = 6.03; 95% CI = 1.14,31.98), living condition (AOR = 13.29, 95% CI = 5.14, 34.35); WHO stage (AOR = 6.06; 95% CI = 2.49,14.74) and ART status (AOR = 7.55; 95% CI = 3.24,17.59) have significant association with prevalence of intestinal parasite.

**Conclusion:**

The overall prevalence of IP was differ by ART status and opportunistic parasite like *cryptosporidium spps* were found in low CD4 counts in ART naive patients. This study identified some environmental and some clinical finding as determinant factor for IP infections. Therefore, public health measures and adherence to ART should be strengthened to improve the quality of life of these patients.

## Background

Intestinal parasitic infections which are caused either by protozoa or helminths or both are among the most widespread of human infections worldwide. It is estimated that as much as 60% of the World’s population is infected with intestinal parasites which may play a significant role in morbidity due to intestinal infections [1]. The rate of infection is also remarkably high in Sub-Saharan Africa, where the majority of Human Immune Deficiency Virus (HIV) /Acquired Immunodeficiency Syndrome (AIDS) cases are concentrated where factors including poverty and malnutrition could promote transmission of both infections in the region [2].

Like in many other developing countries, intestinal parasites are widely distributed in Ethiopia largely due to the low level of environmental and personal hygiene, contamination of food and drinking water that results from improper disposal of human excreta [3]. Intestinal parasites as a major concern in most developing countries have been pronounced with the co-occurrence of malnutrition and HIV/AIDS. Opportunistic parasitic infections are a common feature in HIV/AIDS infections where almost 80% of AIDS patients die of AIDS-related infections including intestinal parasites rather than of the HIV infection itself which usually occur late in the course of HIV infection when Cluster of Differeation (CD4) + T-cell count has been severely depleted mostly below 200cells/mm3 [4-6].

In the absence of Anti Retroviral Therapy (ART) HIV/AIDS patients in developing countries unfortunately continue to suffer the consequences of opportunistic parasites [7]. Patients enrolling into ART programmes with very low CD4 cell counts have heightened risk of morbidity and mortality before ART [8]. There is evidence that the control of these opportunistic parasitic infections in HIV-positive persons under HAART is also induced by the inhibition of the aspartylprotease of the parasites and by the reconstitution of the immune system of the patient [9,10]. However, patients in resource limited settings typically start ART programmes with advanced symptomatic disease and very low blood CD4 cell counts which predisposes them to high rates of both clinical and subclinical opportunistic infections [8]. Therefore, this study was aimed to assess the prevalence of intestinal parasites and associated risk factors in both pre-ART and ART patients in Dessie hospital.

## Materials and methods

### Study design, period and area

A comparative cross-sectional study was conducted from February 1 to April 30, 2012. The study was carried out in Dessie Hospital (DH) ART clinic, South Wollo zone of Amhara regional state, Northeast Ethiopia. Dessie Hospital is found in Dessie town with a distance of 400 km from the capital city of the county, Addis Ababa. According to the 2007 population and housing census, the town had total population of 151,094 and among these, 72,891 was males and 78,203 were females [11]. Dessie Hospital was founded in 1962 and has 17 departments with 300 beds. The Hospital is giving service for 14,114 pre-ART and 10,484 on-ART HIV/AIDS patients.

### Sample size and sampling techniques

Epidemiological Information (EpiInfo) soft ware was used for sample size determination and it gave a sample size of 136 for pre-ART and 136 for on-ART with total sample size of 272 by taking proportion from previous study: Proportion of pre-ART (p1) = 43.5%, Proportion of on-ART (p2) = 24.5%, Odds =2 [12] for pre-ART and on-ART respectively, Marginal error (w) = 5%.

A systematic random sampling technique was used by considering Dessie hospital ART clinic on average gave ART service for 25 pre-ART and 75 for ART adults per day. Since the sample collection period for this study was for two months, the total amount of adult pre-ART and on-ART HIV/AIDS patients that came to the hospital for service was 1500 (N1) and 4500 (N2) respectively and to determine K, the following formula was used, K1 (for pre-ART) = N1/n1 = 1500/136 = 11, so every 11^th^ pre-ART adult HIV/AIDS patients that came to the ART clinic from January to March was included in the sample until the required sample was achieved. Similarly K2(for on-ART) = N2/n2 = 4500/136 = 33 for on-ART adult HIV/AIDS patients, so every 33^th^ on-ART adult HIV/AIDS patients came to the ART clinic from February to March was included in the sample until the required sample was achieved.

### Operational definitions

Pre-ART patients: The person who were found HIV positive but not eligible for ART.

ART patients-The person who were found HIV positive, eligible and started ART already.

Good living condition-low degree of crowding, tape water supply, proper disposal of excreta and cemented and finished type of floor.

### Data collection and processing

A pre-tested structured questionnaire was utilized to collect socio-demographic characteristics, clinical information and other risk factors. The questionnaires were pretested and validated before two weeks in the study time in Selam private Hospital in Dessie on 20 HIV positive patients. A single fresh stool was collected with a labelled stool cup from 272 study participants following standard procedures by laboratory technologist who worked at the ART clinic. A direct saline and iodine wet mount of each sample was used to detect intestinal parasites microscopically. The wet mounts were examined under light microscope at 100 and 400× magnifications [13].

### Formol ether concentration method

A portion of each fresh stool sample was taken and processed. Briefly, 1 g of stool was placed in a clear 15 ml conical centrifuge tube containing 7 ml formalin saline by using applicator stick. The resulting suspension was filtered through a sieve into another conical tube. After adding 3 ml of diethyl ether to the formalin solution, the content was centrifuged at 3200 rpm form 3 minutes. The supernatant was poured away and the tube was replaced in its track. Finally, smear was prepared from the sediment and observed under light microscope with a magnification of 100× and 400× [13].

### Modified ziehl neelsen staining method

A small portion of the fresh stool sample was processed for detection of opportunistic parasites using the Ziehl Neelsen method. Thin smear was prepared directly from sediment of concentrated stool and allowed to air dry. The slides were then fixed with methanol for 5 minutes and stained with carbol fuchsine for 30 minutes. After washing the slides in tap water, they were decolorized with acid alcohol for 1–3 minutes and stained in methylene blue for 1 minute. The slides were then washed in tap water and observed under light microscope with a magnification of 1000X (12).

### Quality control and data analysis

Data collectors were trained and the questioners were pretested before the study time**.** After data collection process, the data were checked for completeness and any incomplete or misfiled questionnaires filed again. Then the result of laboratory examination was recorded on well prepared format carefully and finally attached with the questionnaire. Data were double entered and analyzed by using SPSS-16 database software programme. Descriptive statistics were used to give a clear picture of background variables like age, sex and other variables in well-structured questionnaire. The frequency distribution of both dependent and independent variables were worked out and the association between the independent and dependent variables were measured and tested using OR and 95% CI. The relative contribution of each selected variables to the outcome of interest were assessed using logistic regression.

### Ethical consideration

Ethical clearance was obtained from University of Gondar College of Medicine and Health Sciences School of Biomedical and Laboratory Sciences ethical clearance committee. Permission to conduct the study was also obtained from Dessie Hospital ART clinic. Additionally, after explaining the importance, purpose and procedure of the study briefly a written consent was obtained from study participants. Anyone not willing to take part in the study had full right to do so and confidentiality of the study participants was also maintained. Any study participant who was positive for intestinal parasite was referred to physicians for treatment.

## Results

### Socio-demographic characteristics of the respondents

A total of 272 study participants were included for the analysis of this study. Out of these, 136 were pre-ART (group 1) and 136 were on-ART (group 2). Comparatively, in the Pre-ART and on-ART groups the distribution by sex revealed a predominance of female cases that was 82 (60%) and 78 (57.4%), respectively. The majority of pre-ART (67%) and on-ART (55%) study participants were in the age range of 18–35 years with median age of 32 years (range 18–65 years) and 35 years range (18–63 years); respectively. Majority of pre-ART and on-ART study participants 106 (78%) and 113 (83%) were urban residence; respectively. The assessment of educational status of HIV positives involved in the survey showed 42 (30.9%) of group 1 and 44 (32.4%) of group 2 were able to read and write. Moreover, 44.9% and 46.3% of group 1 and group 2 had <500 birr monthly income, respectively (Table 1).

**Table 1:** Associations of sociodemographic factors of adult HIV patients with prevalence of intestinal parasite by ART status using binary logistic regression in Dessie Hospital ART clinic, Feb.-March 2012 (N = 272)

**Intestinal parasite prevalence**							
**VARIABLES**	**Pre-ART**		**On-ART**		**Total**		**COR (95% CI)**
	**n = 136**		**n = 136**		**N = 272**		
	**Positive**	**Negative**	**Positive**	**Negative**	**Positive**	**negative**	
	**n (%)**	**n (%)**	**n (%)**	**n (%)**	**n (%)**	**n (%)**	
**Age groups**							
18-35 yrs	34(37.4)	57(62.6)	15(20)	60(80)	49(30)	117(70)	1. 26(0. 25, 6.4)
36-53 yrs	17(40.5)	25(59.5)	9(16.1)	47(83.9)	26(26.5)	72(73.5)	1.08(0.21, 5.7)
54-65 yrs	2(66.7)	1(33.3)	0(0)	5(0)	2(25)	6(75)	1*
**Sex**							
Male	20(37)	34(63)	12(20.7)	46(79.3)	32(28.6)	80(71.4)	1*
Female	33(40.2)	49(59.8)	12(15.4)	66(84.6)	45(28)	115(72)	0.98(0.57, 1.7)
**Residence**							
Urban	41(38.7)	65(61.3)	14(12.4)	99(87.6)	55(25)	164(75)	1*
Rural	12(40)	18(60)	10(43.5)	13(56.5)	22(41.5)	31(58.5)	2.12(1.13,4.0)**
**Marital status**							
Single	22(42.3)	30(57.7)	4(9.3)	39(90.7)	26(27.4)	69(72.6)	0.93(0.53,1.62)
Married	31(36.9)	53(63.1)	20(21.5)	73(78.5)	51(29)	126(71)	1*
**Educational Status**							
Illiterate	1(33.3)	2(66.7)	2(16.7)	10(83.3)	3(20)	12(80)	1.44(.28, 7.5)
Read and write	19(45.2)	23(54.8)	6(13.6)	38(86.4)	25(29.1)	61(70.9)	2.36(.74,7.51)
Primary education	16(37.2)	27(62.8)	10(29.4)	24(70.6)	26(33.8)	51(66.2)	2.93(.92, 9.37)
Secondary education	13(38.2)	21(61.8)	6(18.2)	27(81.8)	19(28.4)	48(71.6)	2.28(.69,7.46)
College and above	4(28.6)	10(71.4)	0(0)	13(100)	4(14.8)	23(85.2)	1*
**Occupation**							
Gov’t employer	13(38.2)	21(61.8)	6(19.4)	25(80.6)	19(29.2)	46(70.8)	1*
Merchant	8(42.1)	11(57.9)	0(0)	9(100)	8(28.6)	20(71.4)	.97(0.36,2.58)
Farmer	10(58.8)	7(41.2)	9(42.9)	12(57.1)	19(50)	19(50)	2.42(1.06,5.56)**
Student	1(33.3)	2(66.7)	0(0)	2(100)	1(20)	4(80)	.61(0.06,5.78)
Daily laborers	11(45.8)	13(54.2)	3(12)	22(88)	14(28.6)	34(71.4)	.97(0.43, 2.19)
House wife	4(36.4)	7(63.6)	2(10.5)	17(89.5)	6(20)	24(80)	.61(.21,1.72)
Others	6(21.4)	22(78.6)	4(13.8)	25(86.2)	10(17.5)	47(82.5)	.52(.22,1.2)
**Income**							
<500 birr	25(41)	36(59)	7(11.1)	56(88.9)	32(25.8)	92(74.5)	.93(.43-2.02)
500-1000 birr	18(35.3)	33(64.7)	15(28.3)	38(71.7)	33(31.7)	71(68.3)	1.24(.57, 2.7)
>1000 birr	10(41.7)	14(58.3)	2(10)	18(90)	12(27.3)	32(72.7)	1*

### Prevalence of intestinal parasite in pre-ART and on-ART adult HIV/AIDS patients

A total of 272 stool samples from both groups were examined for intestinal parasitic infections. The overall prevalence of IP in pre-ART was 39% and from these the prevalence of protozoan, helminthic and both protozoan and helminths were 31%, 7.4% and 0.7%; respectively. The prevalence of opportunistic intestinal parasites were 2.2% and from these 1.5% for *Cryptosporidium* spps followed by *I. belli* 0.7%. The most prevalent protozoan parasites and helminths in ART naïve patients were trophozoite of *E.histolytica/dispar* and *A.lumbricoides* with respective prevalence of 19.1% and 2.9% (Table 2).

**Table 2:** Prevalence of intestinal parasites in HIV positive patients with regard to their ART status in Dessie referral Hospital ART clinic, February1 –March 30, 2012(N = 272)

**Parasite identified**	**ART status**		**X**^**2**^	**-value**
	**Pre-ART**	**On-ART**		
	**No (%)**	**No (%)**		
Tro.*E.histolytica/dispar*	26(19.1)	7(5.1)	12.450	<0.00001
Tro.*G.lamblia*	13(9.6)	6(4.4)	2.773	0.096
Cyst of *E.histolytica*	5(3.7)	5(3.7)	0	Nd
Cyst of *G.lamblia*	1(0.7)	0(0)	1.004	0.50
*I.belli*	1(0.7)	0(0)	1.004	0.50
*Cryptosporidium spps*	2(1.5)	0(0)	2.015	0.49
*A.lumbricoides*	4(2.9)	3(2.2)	0.147	1.0
*T.tricuria*	1(0.7)	1(0.7)	0	1.0
*E.vermicularis*	0(0)	1(0.7)	1.004	0.50
*S.stercolaris*	1(0.7)	1(0.7)	0	1.0
*Taenia spps*	3(2.2)	1(0.7)	1.015	0.62
*H.nana*	2(1.5)	0(0)	2.015	0.49
*S.mansoni*	1(0.7)	0(0)	1.004	0.5
protozoan	42(31)	17(12.5)	16.435	0.001
Helminths	10(7.4)	7(5.1)		
protozoa + helminths	1(0.7)	0(0)		
Total	53(39)	24(17.6)	15.235	0.00001

(X^2^) = Pearson chi-square test, nd = not determined.

The overall prevalence of IP among on-ART was 17.6% and from these the prevalence of protozoan, helminthic was 12.5% and 5.1%, respectively. But none of the ART patients were indentified with opportunistic parasites and mixed infection (both protozoan and helminthic infection). The most prevalent protozoan parasites and helminths among ART patients were trophozoite of *E.histolytica/dispar* and *A.lumbricoides* with respective prevalence of 5.1% and 2.2%. The overall prevalence of IP in the pre-ART (39%) was higher when compared to ART groups (17.5%) indicating statistically significant decrease of intestinal parasite in ART patients (p < 0.001) (Table 2).

### Association of intestinal parasite with CD4 among pre-ART and on-ART adult HIV/AIDS patients

The study participants who were pre-ART consisted of 51(37.5%) with CD4 count > 500 cells/mm^3^, 56 (41.2%) with CD4 count 200–500 cells/mm^3^ and 29 (21.3) patients with CD4 count < 200 cells/mm^3^. All the three opportunistic parasites were found in ART naive patients <200 cells/mm^3^ CD4 T-cell counts and from these all *Cryptosporidium spps* infections were significantly associated with < 200 cells/mm^3^ CD4 T-cell counts (p = 0.024).

The study participants who were on-ART consisted of 47 patients (34.6%) with CD4 count > 500 cells/mm^3^, 72 patients (52.9%) with CD4 count 200–500 cells/mm^3^ and 17 patients (12.5%) with CD4 count < 200 cells/mm^3^. Among the 17 on-ART patients with CD4 count < 200 cells /μl, parasites were identified in 9 (52.9%) patients which was statistically significant associated with < 200 cells/mm^3^ CD4 counts (p = 0.0001) (Table 3).

**Table 3:** Prevalence of intestinal parasite among pre-ART individuals (n = 53) and on-ART HIV positive individuals (n = 24) in relation to their CD4 counts in Dessie referral Hospital ART clinic, 2012

	**Pre-ART**				**On-ART**			
**Parasite identified**	**CD4 category**			**p-value**	**CD4 category**			**p-value**
	**>500**	**200-500**	**<200**		**>500**	**200-500**	**<200**	
	**Cell/mm**^**3 **^**no (%)**	**Cell/mm**^**3 **^**no (%)**	**Cell/mm**^**3 **^**no (%)**		**cell/mm**^**3 **^**no (%)**	**cell/mm**^**3 **^**no (%)**	**cell/mm**^**3 **^**no (%)**	
Tro.*E.histolytica/dispar*	7(26.9)	9(34.6)	10(38.5)	.057*	2(28.6)	2(28.6)	3(42.9)	.042**
Tro.*G.lamblia*	2(15.4)	4(30.8)	7(53.8)	.009*	0(0)	0(0)	6(100)	<.001
Cyst *G.lamblia*	1(100)	0(0)	0(0)	.432	0(0)	0(0)	0(0)	Nd
Cyst*E.histolytica/dispar*	5(100)	0(0)	0(0)	.013	4(80)	1(20)	0(0)	.090**
*I.belli*	0(0)	0(0)	1(100)	.156**	0(0)	0(0)	0(0)	Nd
*Cryptosporidium spps*	0(0)	0(0)	2(100)	.024**	0(0)	0(0)	0(0)	Nd
*A.lumbricoides*	1(25)	3(75)	0(0)	.334**	1(33.3)	2(67.7)	0(0)	.78**
*T.tricuria*	1(100)	0(0)	0(0)		3(100)	0(0)	0(0)	.39**
*E.vermicularis*	0(0)	0(0)	0(0)	Nd	0(0)	0(0)	1(100)	.03**
*S.stercolaris*	0(0)	1(100)	0(0)	.487**	0(0)	1(100)	0(0)	.64**
*Teania spps*	1(33.3)	1(33.3)	1(33.3)	.875**	0(0)	1(100)	0(0)	.64**
*H.nana*	0(0)	2(100)	0(0)	.235**	0(0)	0(0)	0(0)	Nd
*S.mansoni*	0(0)	1(100)	0(0)	.487**	0(0)	0(0)	0(0)	Nd
Protozoan	14(27.5)	11(19.6)	17(58.6)	.014*	6(12.8)	3(4.2)	8(47.1)	.001*
Helminths	3(5.9)	6(10.7)	1(3.4)		2(4.3)	4(5.6)	1(5.9)	
protozoa + helminths	0(0)	1(1.8)	0(0)		0(0)	0(0)	0(0)	
Total	17(33.3)	18(32.1)	18(62.1)	.016*	8(17)	7(9.7)	9(52.9)	.00001*

### Associated factors for intestinal parasite among adult HIV/AIDS patients attending ART clinic

Sociodemographic variables in relation to IP prevalence were analyzed by using binary logistic regression model. Place of residence and occupational categories were identified as the major socio-demographic determinants of intestinal parasite among adult HIV/AIDS patients by binary logistic regression. Being rural residence were almost 2 times more likely to had intestinal parasite than those of urban residence (COR = 2.12; 95% CI: 1.13, 3.96) and farmers by occupational categories were almost 2 times more likely to harbour intestinal parasite than government employed (COR = 2.42; 95% CI:1.06,5.56,). However, regarding other socio-demographic characteristics like sex, age group, educational status, monthly income did not show any association with parasite positivity (Table 1).

From the selected environmental and clinical variables as determinant factors for intestinal parasite infection absence of toilet, using river/unprotected water for drinking, having indiscriminate waste disposal system, having contact with animal feaces, having poor living condition, having <200 CD4 counts, being WHO stage III and being Pre-ART were significantly associated with intestinal parasitic infection among adult HIV/AIDS patients in the binary logistic regression with corresponding crude odds ratio of [COR = 6.320; 95% CI: 1.89; 21.191], [COR = 14.2; 95% CI: 3.9, 51.1], [COR = 7.3; 95% CI: 4.04, 13.31], [COR = 3.6; 95% CI: 1.27, 9.92)], [COR = 8.3; 95% CI: 4.52-15.23], [COR = 4.15,95% CI: 1.98,8.72], [COR = 8.4; 95% CI: 3.99, 17.7] and [COR = 2.98; 95% CI: 1.70, 5.22] respectively (Table 4).

**Table 4:** Associations of selected environmental and clinical findings of adult HIV patients with prevalence of intestinal parasite by ART status using binary and multiple logistic regression in Dessie Hospital ART clinic, Feb-March.2012 (N = 272)

**Intestinal parasite prevalence**								
**VARIABLES**	**Pre-ART**		**On--ART**		**Total**			
	**n = 136**		**n = 136**		**N = 272**			
	**Positive**	**Negative**	**Positive**	**Negative**	**Positive**	**negative**	**COR (95% CI)**	**AOR (95% CI)**
	**n (%)**	**n (%)**	**n (%)**	**n (%)**	**n (%)**	**n (%)**		
**Presence of toilet**								
Yes	49(37.7)	81(62.3)	19(15)	110(85)	68(26)	191(74)	1*	1*
No	4(66.7)	2(33.7)	5(71.4)	2(28.6)	9(69.2)	4(30.8)	6.3(1.9,21.2)**	7.6(1.3,44.2)**
**Source of water**								
Tape water	45(35.2)	83(64.8)	18(14)	109(86)	63(25)	192(75)	1*	1*
River/unprotected	8(100)	0(0)	6(66.7)	3(33.3)	14(82.4)	3(17.6)	14.2(3.9651.1)**	6.03(1.2, 31.9)**
**Waste disposal**								
Indiscriminate	26(74.3)	9(25.7)	18(46.2)	21(53.8)	44(59.5)	30(40.5)1	7.3(4.0-13.3)**	
Collected	27(26.7)	74(73.3)	6(6.2)	91(93.8)	33(17)	65(83)	1*	
**Presence of animal**								
Yes	10(45.5)	12(54.5)	7(35)	13(65)	17(40.5)	25(59.5)	1.93(.97-3.81)	
No	43(37.7)	71(62.2)	17(14.5)	99(85.3)	60(26)	170(74)	1*	
**Contact with animal feaces**								
Yes	5(50)	5(50)	4(66.7)	2(33.3)	9(56.2)	7(43.8)	3.56(1.3-9.9)**	
No	48(38.1)	78(61.9)	20(15)	110(85)	68(27)	188(73)	1*	
**Living condition**								
Poor	26(78.8)	7(21.2)	18(47.4)	20(52.6)	44(62)	27(38)	8.3(4.5,15.2)**	13.3(5.14,34.4)**
Good	27(26.2)	76(73.8)	6(6.1)	92(93.9)	33(16)	168(84)	1*	1*
**CD4 categorized**								
<200cell/mm^3^	18(62.1)	11(37.9)	9(52.9)	8(47.1)	27(58.7)	19(41.3)	4.15(1.98-8.72)**	
200-500cell/mm^3^	18(32.1)	38(67.9)	7(9.7)	65(90.3)	25(20)	103(80)	.71(.38-1.33)	
>500cell/mm^3^	17(33.3)	34(66.7)	8(17)	39(83)	25(25.5)	73(74.5)	1*	
**WHO stage**								
Stage I	24(30)	56(70)	5(6.7)	70(93.3)	29(19)	126(81)	1*	1*
Stage II	13(41.9)	18(58.1)	3(7.7)	36(92.3)	16(22.9)	54(77.1)	1.29(.67-2.56)	1.31(.56, 3.06)
Stage III	15(62.5)	9(37.5)	14(70)	6(30)	29(65.9)	15(34.1)	8.4(3.99-17.65)**	6.1(2.49,14.74)**
Stage IV	1(100)	0(0)	2(100)	0(0)	3(100)	0(0)	Nd	Nd
**ART status**								
Pre-ART	53(39)	83(61)	53(39)	83(61)	77(28)	195(72)	2.98(1.7,5.2)**	7.55(3.24,17.6)**
On-ART	24(17.6)	112(82.4)	24(17.6)	112(82.4)			1*	

*Reference category, ** significant association.

Multivariate analysis was adjusted for potential confounding factors such as residence, living condition, absence of toilet, ART status, WHO stage, and CD4. Adult HIV/AIDS patients who do not have toilet in their home were almost 8 times (AOR = 7.57; 95% CI = 1.3, 44.22) more likely to have intestinal parasite than those who have toilet (Table 4). Adult HIV/AIDS patients whose source of water were river/unprotected are almost 6 times (AOR = 6.03; 95% CI: 1.14, 31.97) more likely to be infected for intestinal parasite than those whose source of water is tape water. Concerning living condition those HIV/AIDS patients who had poor living condition are 13 times (AOR = 13.29; 95% CI: 5.14, 34.35) more likely to have parasite than those who have good living condition. Regarding WHO stage those Stage III (AOR =6.06; 95% CI: 2.49, 14.74) patients are 6 times more likely to IP than stage I. With respect to ART status pre-ART patients are 8 times (AOR = 7.55; 95% CI: 3.24, 17.59) more likely to intestinal parasite (Table 4).

## Discussion

This study showed the prevalence of common and opportunistic intestinal parasite among adult HIV/AIDS patients with pre-ART and on-ART in Dessie referral Hospital ART clinic with total prevalence of 39% and 17.6% in pre-ART and on-ART patients; respectively.

The prevalence of intestinal parasite among pre-ART patients was (39%) in this study in line with that of Cameroon study (40.5%), Gondar (43.5%) [12,14]. However; lower in studies Brazil (63.9%), in selected ART centers of Adama, Afar and Dire-Dawa (52%), Arbaminch Chencha and Gideo (45%), in different parts of Ethiopia (57.2%) ( [15-17], unpublished data). This low prevalence in this study might be due to geographic difference in sample size (more than one study area for most reports), considering those patients with, time gap where those studies were done averagely four years ago but nowadays there is a better awareness of the patients about intestinal parasite infection and their cause. They may be diagnosed for parasites by direct wet mount and treated as well.

The prevalence of IP among on-ART patients was 17.6% in this study which was lower than reported in Brazil(24%), Congo (24.6%), Nigeria (30%), in selected ART centers of Adama, Afar and Dire-Dawa (48%), different parts of Ethiopia (42.8%) and Gondar (24.3%) [12,15-19] but it was higher than that of Cameroon (8.5%) [14]. These might be due better follow-up through laboratory tests and better awareness of the patients themselves in adopting prevention and treatment measures against intestinal parasites. Antihelminthics may be given for ART patients for deworming purpose.

This study revealed that statistically significant reduction of intestinal parasite in the ART era (p < 0.001).This finding was in line with previous studies done in Brazil and in different parts of Ethiopia where those studies indicated significant decrease intestinal parasite in the ART era [15-17]. These might be due to the use of HAART with improvement in immunologic conditions of the patients and better response to infections including parasitic ones or even with a direct action on certain enteroparasites, better clinical handling of the patients with constant updating of protocols for treatment and prophylaxis besides their better follow-up through laboratory tests.

This study showed statistical significant difference of *E.histolytica/dispar* between pre-ART and on-ART groups. This is an indication that ART also contributes to the reduction of some non-OIPs even though no evidence that support whether it is the effect of ART or not. However; for other species specific intestinal parasite detected and identified in this study there was no statistically significant difference between the groups and this result was in agreement with study done in Gondar where authors report no statistically significant difference of species specific intestinal parasite between the two groups [12].

The prevalence of *cryptosporidium spps* (1.5%) in pre-ART patients in this study is much lower than Brazil (8.1%), Nepal (31.4%), selected ART centers of Adama, Afar and Dire-Dawa (8%), Nekemet (25%) and that of Gondar (8.7%) [12,15,16,20,21]. The prevalence of *I.belli* (0.7%) in the pre-ART groups in this study is lower than in Brazil (4.8%), Nepal (2.9%), selected ART centers of Adama, Afar and Dire-Dawa (5%) and Nekemet (11.7%) [15,16,20,22]. The existence of such variation may be explained by the difference in geographic location, general hygiene of the population as reported from elsewhere and moreover study participants were recruited without considering their diarrhoea.

This study indicated that all of *cryptosporidium spps* infections among pre-ART groups were found significantly associated with lower <200 cells/mm^3^ CD4 count when compared to the ART experienced patients without this parasite infection in any of CD4 category. This may be due to the fact that opportunistic parasites are known to resolve spontaneously with immune restoration among HIV/AIDS patients on ART [9,10]. The association of these parasite for lower 200 cells/mm^3^ CD4 count was in line with that of Varasani (97.8%), India (83%), in selected ART centers of Adama, Afar and Dire-Dawa (62.5%), in different part of Ethiopia (76.9%) and Hawassa (72%) [16,17,21,23,24].

This study showed that 69.2% of parasite positive HIV/AIDS patients did not have toilet in their home and they were almost 8 times more likely (AOR = 7.566, 95%CI = 1.3- 44.22) to had parasitic infection than those who had toilet in their home. This finding was supported by study done in Nigeria which showed 50% parasite positive patients did not have toilet in their home and also by another study done in Malaysia which showed indiscriminate defecation was significantly associated with parasite positivity with the odds of (OR = 5.01; 95% CI = 3.30–7.62). The higher AOR of this study when compared to Malaysian might be due difference in study population since this study consider HIV/AIDS patients who are immune suppressed but study in Malaysia consider total population [19,25].

Using river water as predictor of IP infection by indicating 82.4% of parasite positive HIV/AIDS were using river water and they were 6 times (AOR = 6.03; 95% CI =1.14, 31.97) more likely to harbour IP than those using tape water. This finding was also supported by study done in Nigeria where 54.6% of parasite infected HIV patients were using river/unprotected water for drinking purpose and by other previous study done in Malaysia which showed that those community residence using river water/untreated water were almost 2 times to had IP infection than those using tape or treated water with the odds ratio of (OR = 2.08; 95% CI = 1.36–3.21). The relatively higher OR of the present study when compared to Malaysian might be due to difference in study population since this study consider HIV/AIDS patients who are immune suppressed but study in Malaysia consider total population [19,25].

This study revealed that poor living condition as one of determinant factor for intestinal parasitic infections by indicating that 62% of parasite positive patients had poor living condition and 13 times more likely (AOR =13.29; 95% CI = 5.14, 34.35) to infected with IP than those with good living condition. The association between poor living condition and intestinal parasite infection was also indicated by previous studies in selected ART centers of Adama, Afar and Dire-Dawa (87.5%) and in different part of Ethiopia (87.6%) of parasite positives were in poor living condition [16,17].

The present finding revealed that being WHO stage III as one of predictor of intestinal parasite infection where stage III patients were 6 times (AOR = 6.06, 95% CI =2.49, 14.74) more likely to have IP than stage I patients and there was study in Nekemet supporting the finding of this study by indicating that the prevalence OIP increased with increasing WHO stage [22].

Considering ART as protective factor those pre-ART patients were almost 8 times (AOR = 7.55; 95% CI =3.24-17.59) more likely to harbour IP than ART experienced patients and this finding was in line with that of Brazil where they indicated those pre-ART patients were almost 6 times more likely to had any intestinal parasite infection than those on-ART patients [15].

In this study, we have done only normal saline, iodine, formol-ether concentration and modified Ziehl-Neelsen staining method for detection of common and opportunistic intestinal parasites. We have not used water-ether sedimentation method for *Microsporidia and* other methods like Molecular techniques and immuno flouscent techniques sensitive for parasites. In addition to this, patients may be diagnosed for parasites and treated as well before. Antihelminthics may be given for deworming purpose. Because of this fact prevalence of intestinal parasites were under estimated in this study.

## Conclusion

The prevalence of intestinal parasites found to be higher in ART naive than attending ART patients. High proportions of intestinal parasites were associated with lower CD4 counts in both pre-ART and on-ART patients. Infections with opportunistic intestinal parasite were associated with lower CD4 counts in ART naïve patients only. Increasing the immune status of HIV infected patients with anti-retroviral therapy may help to reduce acquisition of parasites. Using river/unprotected, absence of toilet in the home, poor living condition, being WHO stage III and being pre-ART significantly increased the prevalence of intestinal parasite in the study area. Public health measures should continue to emphasize the importance of environmental and personal hygiene as well as provide and monitor the quality of drinking water aiming to obtain a better quality of life for those patients. Stool examination should be routinely performed in the follow-up of patients with HIV/AIDS attending ART clinic in order to optimize treatment of institution and other preventive measures. Moreover large scale longitudinal study is needed to determine the effect of ART for both opportunistic and non-opportunistic parasite.

## Competing interests

The authors declare that they have no competing interests.

## Authors’ contributions

AM was the primary researcher, conceived the study, designed, participated in data collection, conducted data analysis, drafted and finalized the manuscript for publication. AA, MD and AA assisted in data collection and reviewed the initial and final drafts of the manuscript. AM, MD, AA and AA interpreted the results, and reviewed the initial and final drafts of the manuscript. All authors read and approved the final manuscript.
